# Human-robot interaction methodology: Robot teaching activity

**DOI:** 10.1016/j.mex.2022.101866

**Published:** 2022-09-21

**Authors:** Anna-Maria Velentza, Nikolaos Fachantidis, Ioannis Lefkos

**Affiliations:** a School of Educational and Social Policies, University of Macedonia, Greece; b Laboratory of Informatics and Robotics in Education and Society (LIRES), University of Macedonia, Greece

**Keywords:** Humanoid robot, Learning outcome, Level of enjoyment, Robot teacher, University, Engineering, Human- robot interaction, Surprise, Familiarity, Teaching, Methodology, Psychology

## Abstract

Research on the use of social robots in education is constantly increasing in the growing field of human-robot interaction (HRI). Consequently, it is essential to determine an appropriate methodology to test how these robots can optimally interact with students. This study specifically looks at how we can use existing knowledge from psychology, neuroscience and educational research and apply them with validity and credibility in HRI studies. We are interested in incorporating research methodologies to evaluate the performance of social robots acting as university professors in a real classroom environment. Moreover, we aim to measure three effects, a) students’ knowledge acquisition (quiz after lecture and final exam grades), b) level of enjoyment (Likert scale questionnaire), and c) level of surprize (analysis of facial expressions filmed by cameras). To identify the relationship between students’ knowledge acquisition, enjoyment, and level of surprize, we designed a series of three experiments, testing three variables: 1. one human-tutor lesson, 2. one robot-tutor lesson, 3. two robot-tutor lessons. In this paper we thoroughly explain the methods used to measuring and testing these variables based on modern and reliable sources.

• Application of Psychological Research Methods to Human-Robot Interaction Studies.

## Graphical abstract

**Figure:**
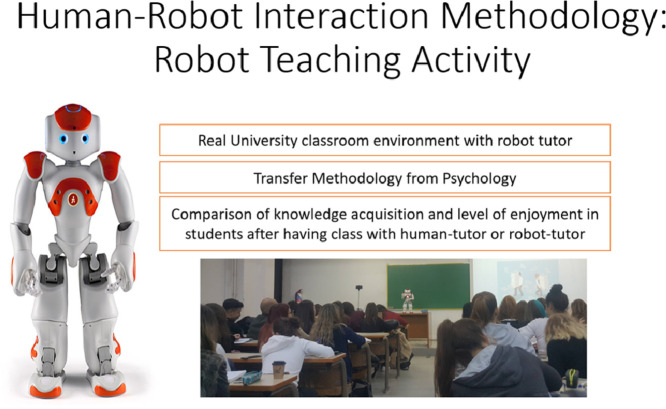

Specifications table

**Table:** 

Subject Area:	
More specific subject area:	*Human Robot Interaction*
Method name:	Application of Psychological Research Methods to Human-Robot Interaction Studies
Name and reference of original method:	Breakwell, G. M., Hammond, S., Fife-Schaw, C., & Smith, J. A. (2006). Research Methods in Psychology. SAGE.
	Herrera, C. D. (2003). A Clash of Methodology and Ethics in `Undercover’ Social Science. Philosophy of the Social Sciences, 33(3), 351–362. https://doi.org/10.1177/0048393103252782
	Hill, C. E., & et al. (2005). Consensual qualitative research: An update. Journal of Counseling Psychology, 52(2), 196–205. https://doi.org/10.1037/0022-0167.52.2.196
	Myers, A., & Hansen, C. H. (2011). Experimental Psychology. Cengage Learning.
	Oatley, P. E. D. of H. D. & A. P. K. (1993). Interpersonal Expectations: Theory, Research and Applications. Cambridge University Press.
Resource availability:	*N.A.*

*Method details [Methodological protocols should be in sufficient detail to be replicated. There is no word limit! You can include figures, tables, videos – anything that you feel will help others to reproduce the method. The main focus of the paper should be on the technical steps required for this method, more than results; where appropriate, guide the reader through the procedure and provide all extra observations or ”tricks” alongside the protocol. Results and Discussion are not sections included in the MethodsX format. However, providing data that validate the method is valuable and required. This section could become a “method validation” paragraph within the Method Details section.]

## Introduction

Many researchers and educators worldwide are using social robots in the classrooms in a variety of applications for different educational levels and age groups. One of the proposed uses for social robots is their employment in place of a tutor or tutor's assistant in the class. In the main research into robots in teaching positions, studies focus on the student's attitudes toward robots and knowledge acquisition [2]. Nonetheless, we are lacking HRI research on how to apply psychological and educational theories in the use of robots as educators. In this study, we propose a protocol in research methodology for using social robots in teaching activities, specifically in a university classroom. We describe and analyse useful steps to compare a) a social robot vs. a human tutor and b) the students’ first-time exposure against the repeated exposure in a robot-tutor university classroom environment, measuring their level of enjoyment and learning outcomes from the course. In our application, we expose university students without engineering backgrounds to one or multiple lectures on fundamental engineering principles with a robot-tutor.

The key points of our methodology are: a) the systematic use of a social robot in the role of a university professor, b) the exposure of students in one course with the robot- tutor for the first time, and/ or more times, c) the measurement of their enjoyment levels and gained knowledge from the course. d)Moreover, the measurement of surprise and familiarity, and finally e) the novel use of undercover researchers to enhance the perceived robot's abilities. The key findings of the current study are thoroughly mentioned in Velentza, Fachantidis, & Lefkos, [40].

There are many techniques on how to use social robots in educational activities. There are more researchers working in this field who are mentioned in the original paper, although in the current section we are focusing on the variety of methodologies and design setups. For example, in the research of [12], the knowledge acquisition and attitudes towards a robot-tutor were investigated against a human-tutor in students aged between 6–16 years old. The students were randomly split into different groups (between groups) having a lecture with either the robot or the human-tutor. The tutors, human, and robot, had the same script, teaching material, slides, and general lesson scenario. The experiment was repeated with different student groups for three times during three consecutive weeks. Likewise, the robot Baxter was used in comparison with a human lecturer to perform storytelling to children, and researchers evaluated their knowledge acquisition by asking them to draw what they remembered from the story [8]. Participants either within or between groups were asked to imagine scenarios with a robot or a human performing the same task and evaluate their overall attitude. Especially in the robot-scenario, they additionally saw different pictures with social robots performing the task [20]. Similarly, Kwok [21], without presenting any interactive activity with any kind of robot, asked elementary school students if the prefer a human or a robot teacher and why in an open type question. The students’ written answers were collected, categorized, and analysed.

Another group of scientists tested the ability of Nao robot to perform as a university professor, by comparing two different teaching styles based on two different mood expressions, a positive and a negative one. The robot provided a variety of capabilities, such as asking questions, to which the students were able to respond with the aid of an MS PowerPoint plugin. They had a short period of time to respond and when the wrong answers outnumbered the correct ones, the robot gave an extended explanation of the question's content. The experimental design was between-groups, and the students were randomly assigned each condition. The same lecture was given twice, once for each condition and the lecture content, i.e. the script, spoken text, and presentation were the same for both conditions, and the students’ location inside the lecture room [45]. Similarly, in another study where the Nao robot performed an educational content storytelling showing different personality styles, the design was between groups and the experimental environment and content of the storytelling remained the same between the different groups [41].

Another technique used in the evaluation of robot-tutors is video analysis. Kennedy and his colleagues compared children learning outcomes after having a lesson with either a screen, an asocial non-personalized robot, or a social personalized robot. 11 students were assigned in each condition, with balanced gender and mathematical skills. Apart from the learning outcome, the conducted behavioural analysis derived from video coding of the children's gaze. One coder completed the analysis for all videos, and a second one verified his/her coding by analysing the 20% of the videos with an average Cohen's Kappa of 0.80 which signifies consequential agreement [19].

The success of a robot-tutor in teaching tasks is usually evaluated based on the student's learning outcome [2]. In several studies, the robot performed the educational activity and researchers evaluated whether the students understand the main concepts of the lesson [4,^14^,29]. In our view, it is important to equally evaluate the students’ learning outcome and level of enjoyment from the course. Educational activities, to be successful, must merge the knowledge acquisition with the element of joy [18].

There are plenty of useful psychological or learning theories regarding the learning process, including techniques, psychological aspects, or even environmental parameters that can enhance or reduce learning acquisition. Nonetheless, there is a gap in research on applying such theories in HRI studies and how to incorporate them into the robot's setup and behavior. In the current study, we are interested in comparing and contrasting the effect of surprise and familiarity -which has been tested in human-human interaction studies (i.e., [11,17,30,33]), in HRI conditions.

## Method details

HRI studies and the use of social robots in the role of a tutor is relatively new and thus, the source of theories to conduct and evaluate experimental conditions and data will be built in the field of Psychology, Social and Learning studies, but also on Neuroscientist findings.

## Experimental studies

*A. How to Compare Human* vs *Robot Tutor in Teaching Activity*

The objective of Experiment I is to compare students’ learning outcome, enjoyment level, and level of surprise when having a lecture with a robot-tutor or with a human-tutor. The methodology for Human- Robot Interaction studies (HRI) stems from the methodology in psychology and especially from the protocols of experiments with human participants. One of the most significant difficulties, but at the same time, the source of experimental validity, is the control between the conditions. The very high degree of control, fundamental between the different experimental conditions, leads to experimental design challenges  [27].

*a) Participants*

The participants were the typical freshmen classroom at the Educational and Social Policies department, entering university after national exams. We chose freshmen students in their first university lecture, to avoid previous experience biases. We had the chance to evaluate their spontaneous reaction to a new stimulus, which was the university lecture. They had no engineering background, which was also an asset, to teaching them a completely new course. There was a total number of 138 participants. The students’ gender was unbalanced, with 94% women. Despite that fact, it is a typical sample for our country's population among students in education relative studies.

Sampling general guide: The participants of an experiment or in other words the sample, is one of the most important factors of accuracy. There are many useful textbooks that can help us choose the sampling method that suits in the experimental purpose [36] and also proposed guides specifically for human-robot interaction studies [3]. We should have pre-defined the characteristics of our sample to fit in a) the target population, b) generalization purposes and b) the task needs.

Target population: In the current study, we focused on university students with specific characteristics (no-engineering background, freshmen, educational studies) and thus we chose the sample accordingly. In case we aimed to examine the surprise effect generally in university students we would have to choose among students from different backgrounds, have representatives from all semesters, and also a variation in schools, studies, etc. Moreover, we should have an equal representation of men and women. Sampling characteristics are also important in case we plan to use the experimental data to design a computational model based on each population's behavior, i.e., model the behavior of students under surprise situations [39].

Generalize: The generalization of the research is also an essential factor. It is the process of going from single cases with specific samples to generic ones, making the research findings possible in time-honored traditional culture in science. By following a solid and replicable methodology, a ‘here-and-now setting’ incorporates general laws of emergence [38].

Task needs: The participants should be able mentally and physically to do the task. Some general advice is to have normal or corrected to normal vision or hearing, and native or proficiency knowledge of the tasks’ language.

*b) Design and Procedure Overview:*

The experimental design was among participants, following the recommendation of previous studies mentioned in the Introduction. The human-tutor, performing the lecture on the first day, was the lecturer of the same along with similar subjects for twenty years. The robot-tutor, performing the lecture during the second day, was the Aldebaran Nao V3.3. At the beginning of the lecture, the tutors (robot or human) introduced his/itself and explained the course's guidelines. Subsequently, they taught the lesson about the basic principles of Cryptography. Both lectures lasted for 30 minutes (without counting the time needed by students to fill in the questionnaires). After the end of the lecture, the tutors thanked the audience for their participation in the course and explained to them that a teaching assistant will give them some questionnaires to evaluate their experience during the course. Moreover, they highlighted that the given knowledge acquisition questionnaire would not be marked, and it is going to be used for the tutor's feedback. The teaching assistant first gave them the LQ, the JQ, and the familiarity and demographic questionnaire. There was no time limit for completing the questionnaires, and the answers were anonymous.

**Fig. 1:**
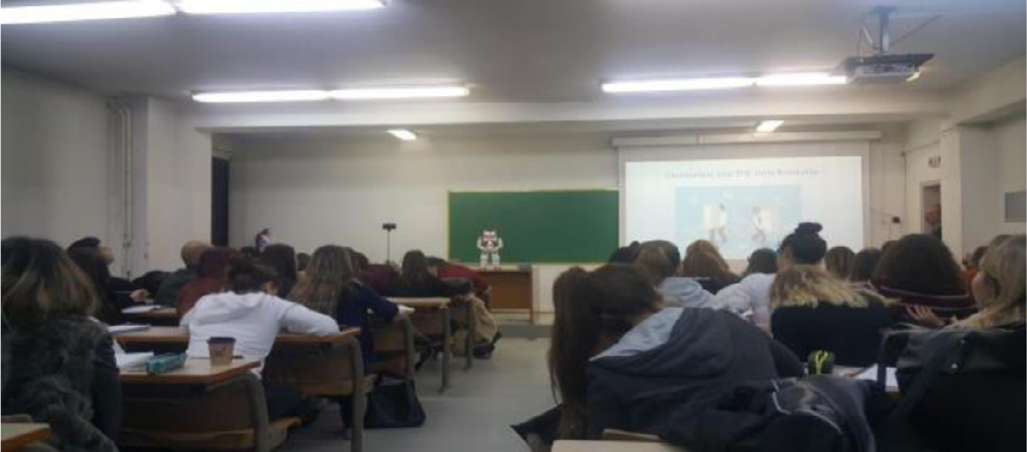
Classroom set up in the robot condition.

There were three cameras placed in front of the class to record the student's reactions during the lecture, as depicted in Fig. 1, one on the right, one on the left side of the room, and one placed on the desk next to the tutor. In all conditions, at the beginning of the lecture, the students were informed about the presence of the cameras, and their rights based on the GDPR protocols, while after the end of the lectures they signed that they agree with the use of their data only for research purposes. Moreover, they were given a one-month period to notify the teaching assistant to dispose of them.

How to control experimental conditions?

We test the effect of a manipulated independent variable. In case we simultaneously vary both a subject or environmental variable and a manipulated independent variable, we will be unable to understand whether effects we are observing. For example, if we gave students who had a course with the robot-tutor their learning questionnaire on a colourful paper and students who had a course with the robot-tutor their questionnaire on a white paper, we would not know whether differences in their learning scores were caused by the nature of the tutor or by the colour of the paper. This complication is known as ‘confounding’ [25].

How to control environmental parameters to keep the same in both conditions:

- Place: Same classroom. By conducting the experiment in the exact same place, we avoid biases from the classrooms’ geometry, i.e., differences in acoustic, students’ distance from the tutor, or design, i.e., distraction decorations, external stimulation, etc.
- Time: Consecutive days, starting and finishing the lecture at the same hour of the day. Avoid circadian rhythm variations [37] or extreme environmental condition changes [13].
- Lighting conditions: The experiment took place in a lecture auditorium without windows (thus no physical lighting) to keep the lighting conditions as much similar in both conditions. We turned on as many lights as needed so that no shadows or light reflections were created from the projector [6].
- Temperature: Experiment I took place the final week of September, and Experiment II in the middle of December. In both cases, we controlled the A/C at the same temperature [13].

For a) practical reasons that will be further explained and b) avoiding expectation biases, we started on the experiment the first day with the human-tutor condition.

How to control independent variable, Tutor's behaviour:

- Content of the lecture: For the first lecture, we choose an engineering subject that does not need prior knowledge to get understandable. The tutor teaches the basic principles of Cryptography. Apart from the theoretic part, with the accompanying PowerPoint presentation, participants saw a short video explaining the analysis and cryptanalysis of the Enigma war machine, and class exercises where participants were given an encryption key and tried to find the encrypted message.
- Script: The script was the same between the conditions. The professor prepared a detailed script in collaboration with the researchers, considering the exact time to pause between the sentences, switches in the PowerPoint presentation slides, and exact time frames to look at them. He rehearsed the script a couple of times and kept a copy on the desk to be able to read from it when needed. The robot's script and movements were prepared based on the script. In some cases, the human- tutor did not follow the script and during the lecture, he added sentences or information. Based on that, we did corrections accordingly to the robot's storytelling.
- Spoken Language: We also took special care for the correct pronunciation of the words in the robot's condition. The lecture was conducted in the Greek language. Although there is a very accurate Greek language version in the robot's software, there were words that needed additional letters or sentences and additional punctuation to avoid uncanny valley effects from the spoken language [7]
- Voice Volume: The voice of the volume was controlled with a microphone placed on the table and two speakers, one on the left and on the right side, to diffuse the sound more smoothly inside the room. Before the experiment, we tested the sound with the aid of other students and lab members, sitting on different seats in the lecture room, verifying at which speakers’ volume they heard the same on the back, the front, and in the middle of the class. The voice volume of the Nao robot was at the highest level. Note that when the classroom is full there is different sound diffusion because there is no echo created in empty halls nor the noise created by the coexistence of many people in the same space.
- Voice Speed: We manipulated the robot's voice speed through the Choreographer, comparing the human-tutor's camera recorded voice with different robot's voice variations. Independent lab members evaluated the variations, and we selected the closer to the tutor's speed one.
- Body Movements: Manipulating the robot-tutor's body movements was the most challenging part. Firstly, at the same time as the writing script, we marked corresponding hand movements for the tutors, i.e., to point or highlight something. Moreover, both tutors had expressive hand movements when talking to the audience. Although we tried to replicate similar movements between the human and the robot-tutor, we did not try to create a robot, replica of the human. The tutors were differentiated in such a way as not to affect the results, but to maintain and utilize the characteristics of their nature (human/robot). We would like them to do the same, but in their own proper (to their nature) way.
- Position of the tutor: Both tutors stood in front of the class. Due to the inconsistency between the robot and human-tutor's height, the robot was placed on a table (which was also in the same position during the human-tutor's lecture), as depicted in Fig. 1.

Avoid Expectation biases: The human-tutor condition preceded the robot-tutor condition to avoid expectation biases [16], [26]. Students during the second day would expect to have a lecture with a robot-tutor. The appearance of a human one would probably disappoint them, distract their attention in case they were looking for what their classmates told them, and create a feeling of injustice, together may lead to systematic errors. This would affect all the given questionnaires (LQ and JQ), and their level of surprise.

According to the aim of each study and thus the environment where the experiment will take place, there are other controls that should be taken into consideration, such as avoiding the interventions (loud noises, unexpected events taking place). For human-robot interaction experiments in social activities, we suggest following experimental design guidelines for social sciences [23].

Going Undercover: Social Scientists usually go undercover when they are afraid of biases, in some cases, they reveal their identity or the purpose of their study. Another case is when they tend to protect the subject/ participants in case of an emergency. The practice of undercover raise a lot of ethical questions and at the same time despite the benefits, it leaves us skeptical of their findings and reports [15]. Going undercover in human-robot interaction studies can be compared with the Wizard of Oz methodology. The participants are tricked into believing that they interact with an autonomous robotic agent. Strazdas et al. implemented a similar approach to present to their subjects a restriction-free, multimodal HRI with all the necessary for their study features such as posture, head pose, speech, etc. [35]. In our case, the participants were freshmen in their first lecture at the university. They did not know their classmates yet and thus, it was not suspicious (did not cause any biases) the appearance of two casually dressed girls (our researchers) mixed with them. On both experimental days, the undercover sat at the exact same spot, one on the left side of the class and one on the right, wearing the exact same clothes. Both the tutors (human and robot) asked them the same question to hear the same answer and give them back feedback. When the tutor asks (relevant to the course subject), many students, including the undercover raise their hands. The tutor refers to the undercover as ‘the girl with the blue t-shirt, sitting in the second row of seats’. The tutor used similar identification for the second undercover in the second question. The identification was specific to: a) avoid having another student answer the question, and b) show intelligence and recognition skills in the robot-tutor condition.

In another study, we used the undercover researchers in the exact same setup [44] in order to present the robot's capabilities in a short time period, including its ability to provide feedback. The method helped us evaluate the future teachers’ attitudes regarding the ideal peer-tutor robot characteristics before and after having a course with a robot-tutor. The undercover researchers set up, is helpful especially in human-robot interaction studies to focus on the cognitive outcome of the interaction without spending time on costly or time-consuming implementations.

Things to take into consideration: The undercover should:

• Not cause any attention from the participants.
• Act, behave and dress similarly to the participants.
• Have the exact same appearance, script, and behaviour between conditions to avoid systematic error or adding an unwillingly extra parameter in the experiment.

*c) Measurements*

Learning quiz (LQ): The total number of questions was ten, enough to draw a safe conclusion without discouraging participants from answering them. There were five Multiple Choice Questions (MCQ) with only one correct answer with four multiple answers per question, followed by five open questions.

Type of the questions: We used both types of questions since multiple-choice and open-ended questions correspond to different aspects of comprehension processes [28]. Multiple-choice questions are linked with the recognition process, since both the questions and the answers serve as retrieval cues. On the other hand, open-ended questions are linked with the recall process since participants have fewer cues and produce the information from their memory. There are two stems involved in the recall process, firstly, generate an answer and determine whether it seems correct. The recognition process, used on a multiple-choice test, only involves one step, to determine which possible answer from the listed ones seems most correct [34]. In the current study we could not time the participants' answers because we gave the questionnaires at the same time in a large sample, and in paper form. Although, in cases where timing is plausible, it is important to consider that the response time for the open-answer questions includes the time to read the question and type an answer. On the hand, the response time for the multiple-choice questions includes the time to read the question and the listed options and to select the more appropriate answer [32]. We recommend the timing for the multiple-choice questions and not for the open-answer questions. Slower reaction time is linked with a higher level of attention, due to the fastest recall. Timing in open-ended questions also incorporates the participants’ writing speed, which should be controlled.

Content of questions: The questions were about the content of the lecture. In our previous studies [41,43] before giving a learning or memory questionnaire after a robot's or human's storytelling we run a pilot study to evaluate the questions’ quality, especially for the multiple-choice questions. Ideally, we create at least twice as many as we intend to include in our questionnaire. A sample, with similar characteristics to the targeted sample, hears the storytelling from a recording and answers all the questions. There is no need to replicate the whole experiment because our target is to evaluate the questions and not the robot's appearance or any other aspect of our experiment. In this pilot study, the storytelling serves as the independent variable and the questions as the dependent. The questions should not be very easy -correctly answered by almost everyone- nor very difficult -correctly answered by almost none-. A fair rage is a response rate of <30 and >80%. It is also important to make sure that all wrong answers (distractors) are plausible. This method is also strongly recommended for experimental setups where the memory test is used to measure participants’ level of attention.

In the current study, we did not follow this procedure. It was a real-life educational activity with specific requirements from the course's lecturer on what he considers important for students to remember from the lesson, and thus we created the questions based on them.

Marking: The LQ was marked by the teaching assistant who was teaching the course together with the professor for already three years, with an exam marking scheme, to avoid biases from the tutor who may manage to relate the questionnaires with the experimental condition.

Level of Enjoyment Questionnaire (JQ): We used a 35 items Likert scale questionnaire to evaluate students’ enjoyment level from the course. The questionnaire has been formed and used by Velentza, Heinke, Wyatt [42], and was given to participants after witnessing a robot's storytelling, to evaluate their enjoyment level from the interaction. We had all the rights to use the JQ questionnaire. The questionnaire fitted our purposes due to a) its content (previously used for similar purposes), and b) the short wording of the questions (There were 35 single words with both positive, and negative meanings i.e., Interesting, Ugly, Inspirational, etc. and participants had to evaluate their experience from 1: Strongly Disagree to 5: Strongly Agree.).

Familiarity Questions: We designed a short questionnaire with seven Likert scale statements to evaluate students' familiarity with a) technology, b) robots, and c) the subject of the course. The questions were straightforward to what we were interested in evaluating, i.e., ‘have you ever used a robot’, ‘do you have IoT-related knowledge’, etc.

Demographic characteristics: The demographics we were interested in were the students’ gender and age. Those questions were placed at the end of the final questionnaire [5] to avoid participants' possible feelings of awkwardness, skepticism, or prejudice toward the study.

Tips: The number of questions in all the questionnaires should be large enough to draw a safe conclusion, but small enough to be answered by as many participants as it can without getting them bored (possibility to answer randomly just to finish the task) or discouraging them.

Code the questionnaires: All the questionnaires were anonymous, and after they got collected, they were packed together and marked with a unique code per person. The researcher who coded them was able to understand from which condition each questionnaire was collected, while the person who marked them was not able to retrieve that information.

Level of surprize: We measured the participants’ level of surprise based on their facial expression analysis conducted based on video recordings. We used three cameras, all of them placed on the front side of the lecture room. Likewise, we strongly recommend the use of an additional camera at the back of the room, to show the lecturer, for internal evaluation, future reference, and dissemination purposes.

*d) Data Analysis*

LQ scores: The total number of correct answers per person was compared between the two conditions. For the analysis of the correct answers, we applied a paired sample t-test [22].

JQ scores: In case we aim to compare the JQ scores from human and robot-tutor conditions, a t-test would be appropriate. However, in our case, we also needed a multiple comparisons test to stress the JQ scores of the first experiment (human vs robot tutor) with the JQ scores of the second experiment (first time vs second-time robot tutor). Thus, to find if there were any significant differences between the different conditions from the JQ responses, we handled a between-participants Bonferroni multiple comparison test [9].

Furthermore, based on experimental research findings [24], the highest learning outcome does not mean that the students enjoyed the learning process equally, and thus, we investigated whether the experimental condition that leads to higher LQ scores, equitably leads to high scores in JQ with the use of an ANOVA test.

Additionally, we handled a Pearson r correlation analysis, between the LQ, JQ, familiarity, and demographic scores for both robot and the human condition. All the analyses were performed with the aid of SPSS 25.

Familiarity data: Since we designed the questions based on the experimental needs, we performed a factor analysis to split the questions into groups. The Factor Analysis led us to 3 categories. For each category, we report the Cronbach's alpha reliability measure: 1) use of technology in education (α=0.85), 2) familiarity with the course's subject (α=0.89), 3) familiarity with robots (α=0.91).

Video analysis to measure the surprise effect: The camera recordings were muted, to avoid revealing the tutors’ nature, and were analyzed based on the students’ facial expressions by an independent cognitive psychologist. Based on evolutionary emotion psychologists and neuroscientists, the emotion of surprise is detectable through pupil dilation, skin conductance increase, head movements, dropped jaw, not drawn together raised eyelids and eyebrows and hands that are brought to the face like a shield [30]. The analysis was conducted with the use of ELAN 5.9 Software [1]. The analysis started with the tutor's first welcome to the students and finished when the teaching assistant started giving the questionnaires to the students. We created one tier per participant only for students who were fully visible during the video recording, in which we marked the milliseconds during which a surprise motion appeared. To measure the surprise effect, for all the tiered participants per condition, we summed up the marked milliseconds and divided them by the total number of participants, to end up with one number, the average milliseconds of surprise per condition. Finally, we conducted a t-test between the different conditions.

*B. Experiment II: How to Compare Robot Tutor for the first-time* vs *Robot Tutor for the same time in Teaching Activity*

The objective of Experiment II is to compare students’ learning outcomes, enjoyment level, and level of surprise when having a lecture with a robot-tutor for the first time or with a robot-tutor for the second time. We expect that students who experience the robot-tutor lecture for the first time will have lower learning outcome in comparison with those who experience the lecture for a second time, due to their expected high level of surprise. Students who experience the robot-tutor lecture for the second time are expected to increase their familiarity with the tutor without losing the whole surprise effect from its appearance, and thus we expect them to be more motivated [30] with more correct answers in the learning quiz in comparison with the other group.

*a) Participants*

The students who participated in Experiment II, are the same who participated in Experiment I. Those who experienced the human-tutor lecture in Experiment I, participated in the robot-tutor condition for the first time in Experiment II and those who experienced the robot-tutor lecture in Experiment I participated in the robot-tutor condition for the second time. The lectures took place ten weeks after the first lectures, the final semester week before the Christmas break. Due to the festive season and the fact that many students booked their tickets to travel home earlier, the same was smaller, N=37 had a lecture with the robot-tutor for the second time, and N=52, had a robot-tutor lecture for the first time, N=52. Based on that, we strongly recommend avoiding the final week before breaks for research with university students, especially for test-re-test procedures or generally cases that require the same sample.

*b) Design and Procedure*

The experimental design and procedure mimicked the robot-tutor condition from Experiment I and was identical for the two conditions, first time and second-time robot-tutor. The lecture's content was more sophisticated than the first experiment, take the courses’ timetable, and it was about hardware, internal and external systems, storage devices of a computer, and social issues about technology such as technological illiteracy. The lecture lasted for 30 minutes.

*c) Data Analysis*

We used the same data analysis techniques since we had the same measurements. For the video recordings, we proceeded to small adjustments before sending them for analysis. Although the timetable remained the same for the students, it was possible for some of them to change the course attendance day without official notice. Thus, at the beginning of the course, the robot-tutor asked the students who were enrolled for a different day to raise their hands. More specifically, in the group where the students were about to have their second robot-tutor lecture, the robot asked those who ‘had never had a course with a robot’ to raise their hand. Similarly, in the group where the students were supposed to have their first robot-tutor lecture, the robot asked those who ‘had experienced a robot-tutor lecture’ to raise their hands. The psychologist was informed to exclude them from the group analysis and transfer their data analysis to the other group. Moreover, to avoid analysing their LQ and JQ scores with the wrong group, we added a question in the demographic questionnaire, asking if it was their first or second time having a course with the robot-tutor during this course. Those questionnaires were transferred and grouped correspondingly.

*C. Comparison of Experiment I* vs *Experiment II*

We can extract interesting points when comparing the results from Experiments I and II. There are some measurements that can be directly compared with the use of appropriate statistical analysis, such as students’ level of enjoyment and surprise, while others can only be used for observation or demonstrating a trend, such as the learning outcome scores.

The level of students’ enjoyment can be measured and compared between the four conditions: human-tutor and robot-tutor from the first experiment and first- and second-time robot-tutor from the second experiment by the Bonferroni analysis. One major point for validating purposes is to find similar results among students who experienced a robot-tutor lecture for the first time between the two experiments.

The LQ scores cannot be directly compared between the two experiments since the lessons were on different subjects. Although, we can observe the student's level of knowledge acquisition by comparing the percentages of correct answers per condition. This comparison can lead to a trend if students pay more attention to the lecture when they are familiar with the tutor.

Students’ level of surprise can be directly compared with the same group's average surprise time between different conditions, i.e., students’ average surprise time when having robot-tutor lecture during Experiment I and when having a second robot-tutor lecture during Experiment II. It is also expected that the average surprise time between those who had their first robot-tutor lecture in Experiment I and II to be similar. However, there are some additional parameters that may affect this outcome, such as the familiarity with the course subject after ten weeks of lectures, and expectation biases. It is very highly possible that students who experienced the robot-tutor lecture in Experiment I informed their classmates who would probably expect to have a similar lecture, formulate an opinion based on the descriptions, or even felt underprivileged for not having such a lecture. All those possible factors may remove some surprise elements.

In conclusion, the comparison between Experiment I and II can demonstrate under which conditions students paid more attention to the tutor, and under which circumstances they find the lecture more interesting, inspirational, and, generally, how they evaluated their experience when the tutor was a conventional human-professor in comparison with a robot.

## Experiment III: EXAM DAY

To evaluate the long-term learning outcome of the robot-tutor, we expanded the experiment to the day of the final exams. The more effective and unbiased way to measure long-term learning outcomes would be to give them a knowledge acquisition without noticing them first. However, our target was to evaluate both students’ learning outcomes and *motivation*. The students' results in final exams are a combination of various factors [10] such as class attendance, conscientiousness, verbal ability, and motivation to succeed. Thus, the evaluation of final exam scores is not a reliable and valid way to measure students’ learning outcomes, but it can be used as an indication of the motivation that can be caused by a robot-tutor lecture.

*a) Participants*

The students were registered for administrative purposes to give the exam in different groups according to the first letter of their surname, every one hour on the same day. In each group, they were mixed, however, we categorised them into three groups (a) those who attended one robot-tutor lesson, N= 78 (b) two lessons N= 60, (c) never, N=64.

Those who are included in the third group are students who were missing from the lecture on the days of the experiments.

*b) Procedure*

The final exam for passing the course was developed through a university software platform and was held in the University's pc lecture room. Based on the LQ scores per question, per experiment, we added the questions with the less correct answers -the more difficult ones- to the total number of exam questions.

*c) Data Analysis*

To analyse the data, we used only the six questions from the LQ, and we applied the Fisher's Least Significance Difference Test (LSD) which is a powerful post hoc comparison for three groups [31].

The results from all the data analyses are thoroughly reported in Velentza, Fachantidis, & Lefkos, [40].

## General discussion and conclusions

The present study focused on the human-robot interaction methodology between university students without engineering backgrounds and a robot-tutor. The used methodology basically stems from well-established techniques and protocols from the fields of psychology, educational studies, and neuroscience. We were interested mainly in how the robot-tutor can improve the first-year university students’ knowledge about basic engineering subjects, in addition to the level of enjoyment from the lecture's experience. The students experienced one or multiple lectures held by a human-tutor and by a robot-tutor, and additionally, we evaluated their level of surprise during the lecture and how this may affect both their level of enjoyment and learning outcome.

First, we analysed how to compare a robot-tutor and a human-tutor performing the same teaching activity based on the participant's learning outcomes, level of enjoyment, and surprise. We mainly focused on how to keep the different conditions as similar as possible, and how to organize the real-life educational task set up, and we also explained the use of undercover researchers.

For the second experiment, we built a robot-tutor lecture similar to Experiment I with different course subjects. We again compared the students’ learning outcome, level of enjoyment, and surprise when they experienced a robot-tutor lecture of the first or for the first or for the second time. We kept the same methodology with Experiment I. Moreover, we explain the appropriate statistical analysis for extracting safe results from the study regarding what we measure in each condition. Finally, we used the final exam grades to evaluate the students’ motivation.

Generally, this study demonstrates that HRI researchers can have a common ground in the human experiments with social robots serving as tutors to students and hope to help them have a clear map to replicate similar studies or find answers to questions like ours.
